# The Effect of Laterality and Primary Tumor Site on Cancer-Specific Mortality in Breast Cancer: A SEER Population-Based Study

**DOI:** 10.1371/journal.pone.0094815

**Published:** 2014-04-16

**Authors:** Jing Bao, Ke-Da Yu, Yi-Zhou Jiang, Zhi-Ming Shao, Gen-Hong Di

**Affiliations:** Department of Breast Surgery, Cancer Center and Cancer Institute, Shanghai Medical College, Fudan University, Shanghai, P. R. China; Health Canada and University of Ottawa, Canada

## Abstract

**Background:**

Reduced overall survival has been observed in patients with left-sided versus right-sided breast cancer due to cardiac toxicity after radiotherapy. However, the effect of laterality and primary tumor site on breast cancer-specific mortality (BCSM) remains unclear.

**Patients and Methods:**

We analyzed data from 305,443 women ages 20- to 79-years-old diagnosed with breast cancer between 1990 and 2009. The data were obtained from the population-based Surveillance, Epidemiology, and End Results (SEER) program of the U.S. National Cancer Institute. The survival outcomes with regard to laterality and primary tumor site were compared using univariate and multivariate (Cox proportional hazards regression model) methods.

**Results:**

In the multivariate analysis, BCSM was affected by the primary tumor site (P<0.0001) but not laterality (P = 0.331). The combined effect was piecewise: using the left upper-outer quadrant as the reference, the BCSM hazard ratio (HR) was not significant in the right upper quadrant (P = 0.755) and the right central portion (P = 0.329). The BCSM HR was slightly increased in the left central portion as well as the left and right lower-outer quadrants (HRs from 1.136 to 1.145; P<0.0001). The BCSM HR was significantly increased in the upper-inner and lower-inner quadrants (HRs from 1.242 to 1.372; P<0.0001) on both sides. Laterality only impacted BCSM in patients with breast cancer located in the central portion (HR, 1.100; P = 0.013, using the right side as the reference).

**Conclusion:**

Patients with tumors in the upper-outer quadrant of both sides and the right central portion have a better prognosis than patients with tumors at other locations. Laterality is not regarded as a prognostic factor in breast cancer.

## Introduction

In-depth studies in the field of breast cancer preferably describe breast cancer using various characteristics, such as age, histologic grade, local tumor size, regional lymph node involvement, presence of lymphovascular invasion, hormone receptors and HER2/*neu* status, given the prognostic value of these factors in breast cancer survival [1]–[3]. Tumor location is not regarded as a universally acknowledged prognostic factor in cancer-specific survival unlike the above indices; however, various studies have concluded that medial and lower sites are related to poor survival [4]–[5]. Moreover, some patients with stage I breast cancer die of their disease due to undetected internal mammary chain (IMC) involvement, which is not routinely investigated in lymph node metastases [5]–[6]. The presence of IMC metastases depends on tumor location in the breast; a higher prevalence of IMC metastases in tumors of the inner quadrants has been noted, especially in the lower-inner quadrant [6]. In this regard, the primary tumor site cannot be neglected in a systemic evaluation of breast cancer.

Since the last decade of the twentieth century, an increased risk of mortality due to ischemic heart disease has been observed in women with breast cancer treated with radiotherapy. Specifically among these patients, an excess of cardiac deaths was noted in left-sided breast cancer compared with right-sided breast cancer [7]–[8]. However, given the improvement of radiotherapy techniques and protective measures, radiation-related mortality has substantially decreased in recent years [9]. Despite these studies, the influence of laterality on breast cancer-specific mortality (BCSM) remains unknown.

To determine whether laterality and primary tumor site independently contribute to breast cancer prognosis, we evaluated the effect of laterality and primary tumor site on BCSM.

## Patients and Methods

### Patient Selection

We collected breast cancer records from the population-based Surveillance, Epidemiology, and End Results (SEER) program of the U.S. National Cancer Institute [10]. The cut-off date of follow-up was November 2012. In total, 305,443 female patients diagnosed with invasive breast cancer between January 1, 1990 and December 31, 2009 were included in the study. In addition, 93,954 patients diagnosed before 1990 were excluded as a result of unavailable hormone receptor data; 39,179 patients diagnosed after 2009 were also excluded due to inadequate follow-up time.

**Figure 1:**
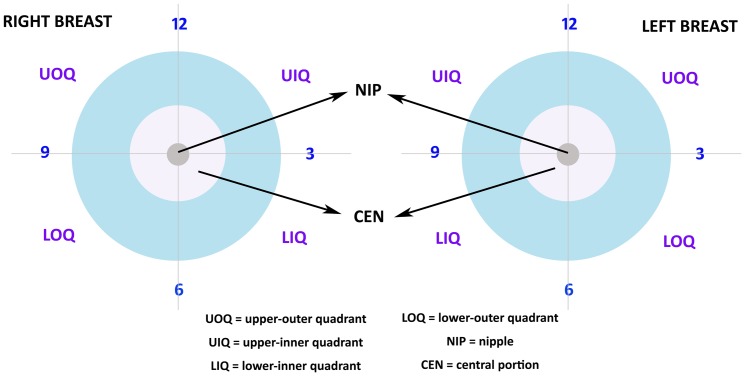
Classification of the Primary Breast Tumor Site. The primary breast tumor site is classified as five groups: upper outer quadrant (UOQ), upper inner quadrant (UIQ), lower inner quadrant (LIQ), lower outer quadrant (LOQ) and central portion (CEN).

**Figure 2:**
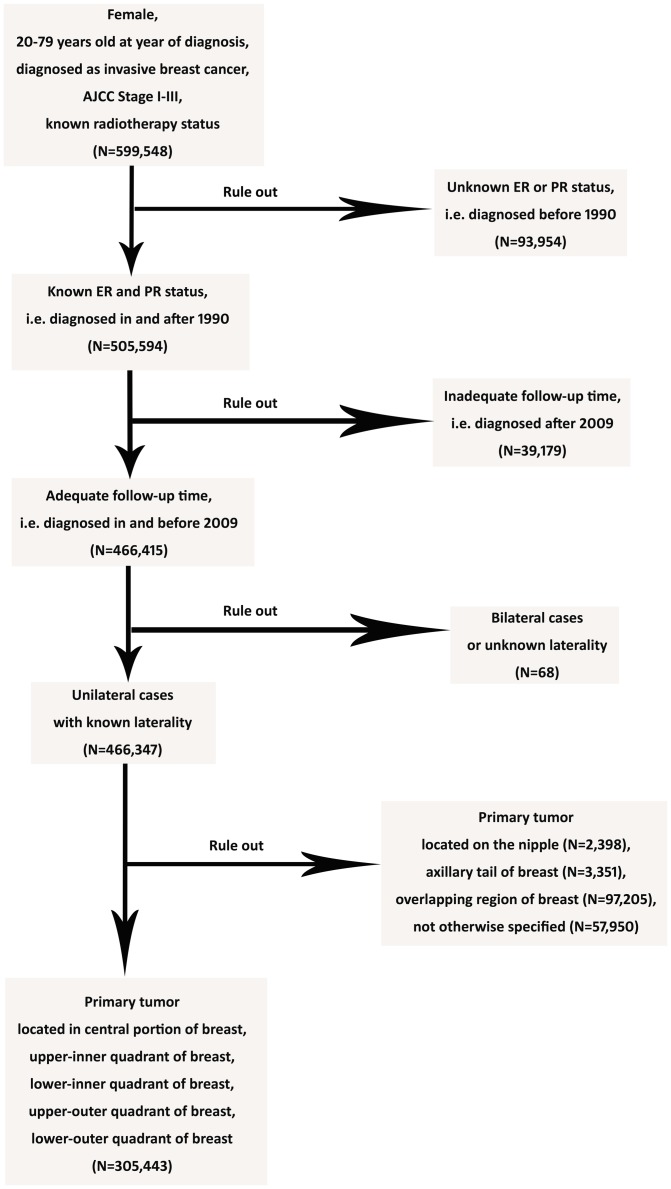
Flow Diagram of Inclusion Criteria and Exclusion Criteria. The inclusion criteria are presented as follows: female sex, between 20–79 years at age of diagnosis, diagnosed as invasive breast cancer between 1990 and 2009, AJCC stages I to III and known radiotherapy status (599,548 patients). 93,954 patients diagnosed before 1990 were excluded as a result of unavailable hormone receptor data, while 39,179 patients diagnosed after 2009 were also excluded due to inadequate follow-up time. Besides, 68 patients with bilateral cases or unknown laterality were excluded. Regarding the primary tumor site, tumors located in the four quadrants and central portion were included, and tumors located on the nipple (2,398 patients), axillary tail (3,351 patients), overlapping region (97,205 patients) and not otherwise specified (57,950 patients) were excluded to avoid selection bias. So, 305,443 patients were included in this study eventually.

The specific inclusion criteria are presented as follows: female sex, between 20–79 years at age of diagnosis, diagnosed between 1990 and 2009, unilateral breast cancer with documented primary site and exclusive laterality, pathologically confirmed invasive breast carcinoma, American Joint Committee on Cancer (AJCC) stages I to III and known tumor size as well as lymph node (LN), estrogen receptor (ER) and progesterone receptor (PgR) status. Regarding the primary tumor site, we included tumors located in the four quadrants and central portion, which is the subareolar area extending 1 cm around areolar complex (Figure 1). Tumors located on the nipple (2,398 patients), axillary tail (3,351 patients) and overlapping region (97,205 patients) which indicated a single primary tumor involving two adjacent quadrants of the breast (according to SEER Program Coding and Staging Manual 2013) were excluded to avoid selection bias (Figure 2).

### Outcomes Measures

The primary outcome of the retrospective cohort study was BCSM because BCSM minimizes confounding bias caused by non-breast cancer-related mortality censored at the date of death. Thus, BCSM was calculated as a mortality rate using deaths specifically attributed to breast cancer.

### Statistical Analysis

The study variables included age and year of diagnosis, laterality and the primary tumor site as well as LN, ER, PgR, and radiotherapy status. Furthermore, laterality and the primary tumor site were mutually calculated to determine whether the interaction between these two factors influenced BCSM.

The Chi-square test was used to compare clinicopathological characteristics of the primary tumor site. The survival curves were generated using the Kaplan-Meier method, and the log-rank test was performed to evaluate the survival difference. Adjusted hazard ratios (HRs) along with 95% confidence intervals (CIs) were calculated using the Cox proportional hazards regression model. Pair-wise comparisons were conducted between various combinations of laterality and primary tumor sites to determine BCSM differences. All statistical analyses above were performed using the Statistic Package for Social Science (SPSS), version 19.0 (SPSS Inc., Chicago, IL). Two-sided P values less than .05 were considered statistically significant.

## Results

### Descriptive Statistics

With a median follow-up of 72.0 months, we identified 305,443 eligible patients for our study; of these patients, 24,944 patients (8.2%) died from breast cancer, whereas 36,198 patients (11.9%) died from other causes. Table 1 summarizes the clinicopathological characteristics of patients from the SEER database according to the site of the primary breast tumor. Using the Chi-square test, we found significant differences among the five categories with regard to patient age, year of diagnosis, tumor size, LN involvement, and laterality as well as ER, PgR, and radiotherapy status. Interestingly, tumors located in the central portion were correlated with older age, earlier year of diagnosis, increased tumor size, increased LN involvement, ER positivity, PgR positivity and reduced radiotherapy. In addition, increased breast cancer incidence was observed in the left side and upper-outer quadrant based on laterality and primary tumor site, respectively. However, more tumors in the upper-outer quadrant were located on the right side compared with the left side, whereas left-sided tumors had a slightly higher incidence rate in other categories, especially in the inner quadrants (Table 1).

**Table 1:** Clinicopathologic Characteristics of Breast Cancer Patients from SEER Database by Primary Site.

	No. (%) of Patients						
	Total (n = 305,443)	UO (n = 169,346)	UI (n = 51,303)	LI (n = 26,603)	LO (n = 32,618)	CEN (n = 25,573)	
Characteristics							P
**Median Follow-up (mo) (IUR)**	72.0 (38–116)	74.0 (39–118)	70.0 (36–113)	70.0 (29–114)	69.0 (37–113)	73.0 (38–118)	
**Patient age (y)**							<.0001
**20–39**	18,557 (6.1)	10,741 (6.3)	3,089 (6.0)	1,365 (5.1)	2,140 (6.6)	1,222 (4.8)	
**40–59**	143,867 (47.1)	81,227 (48.0)	24,597 (47.9)	11,814 (44.4)	15,347 (47.1)	10,882 (42.6)	
**60–79**	143,019 (46.8)	77,378 (45.7)	23,617 (46.0)	13,424 (50.5)	15,131 (46.4)	13,469 (52.7)	
**Year of Diagnosis**							<.0001
**1990–1994**	33,601 (11.0)	19,782 (11.7)	4,970 (9.7)	2,683 (10.1)	3,262 (10.0)	2,904 (11.4)	
**1995–1999**	47,100 (15.4)	26,595 (15.7)	7,350 (14.3)	3,995 (15.0)	4,735 (14.5)	4,425 (17.3)	
**2000–2004**	104,619 (34.3)	58,274 (34.4)	17,416 (33.9)	9,201 (34.6)	10,985 (33.7)	8,743 (34.2)	
**2005–2009**	120,123 (39.3)	64,695 (38.2)	21,567 (42.0)	10,724 (40.4)	13,636 (41.8)	9,501 (37.2)	
**Tumor Size**							<.0001
**0–2 cm**	204,014 (66.8)	110,871 (65.5)	35,797 (69.8)	19,461 (73.2)	22,220 (68.1)	15,665 (61.3)	
**2–5 cm**	89,438 (29.3)	51,120 (30.2)	14,012 (27.3)	6,589 (24.8)	9,467 (29.0)	8,250 (32.3)	
**>5 cm**	11,991 (3.9)	7,355 (4.3)	1,494 (2.9)	553 (2.1)	931 (2.9)	1,658 (6.5)	
**LN Status**							<.0001
**Negative**	210,240 (68.8)	112,361 (66.3)	40,089 (78.1)	20,038 (75.3)	21,708 (66.6)	16,044 (62.7)	
**Positive**	95,203 (31.2)	56,985 (33.7)	11,214 (21.9)	6,565 (24.7)	10,910 (33.4)	9,529 (37.3)	
**ER Status**							<.0001
**Negative**	68,085 (22.3)	39,124 (23.1)	11,450 (22.3)	5,942 (22.3)	6,927 (21.2)	4,642 (18.2)	
**Positive**	237,358 (77.7)	130,222 (76.9)	39,853 (77.7)	20,661 (77.7)	25,691 (78.8)	20,931 (81.8)	
**PR Status**							<.0001
**Negative**	99,709 (32.6)	56,484 (33.4)	16,482 (32.1)	8,622 (32.4)	10,467 (32.1)	7,654 (29.9)	
**Positive**	205,734 (67.4)	112,862 (66.6)	34,821 (67.9)	17,981 (67.6)	22,151 (67.9)	17,919 (70.1)	
**Radiotherapy**							<.0001
**Without RT**	139,306 (45.6)	75,267 (44.4)	22,212 (43.3)	12,267 (46.1)	15,294 (46.9)	14,266 (55.8)	
**With RT**	166,137 (54.4)	94,079 (55.6)	29,091 (56.7)	14,336 (53.9)	17,324 (53.1)	11,307 (44.2)	
**Laterality**							<.0001
**Left-sided**	155,126 (50.8)	84,246 (49.7)	26,792 (52.2)	14,169 (53.3)	16,926 (51.9)	12,993 (50.8)	
**Right-sided**	150,317 (49.2)	85,100 (50.3)	24,511 (47.8)	12,434 (46.7)	15,692 (48.1)	12,580 (49.2)	

Abbreviations: UO =  upper outer quadrant of breast; UI =  upper inner quadrant of breast; LI =  lower inner quadrant of breast; LO =  lower outer quadrant of breast; CEN =  central portion quadrant of breast; IUR =  interquartile range; LN =  lymph node; ER =  estrogen receptor; PR =  progesterone receptor; RT =  radiotherapy.

### The Respective Effects of Laterality and Primary Tumor Site on BCSM

Based on multivariate analysis, a significant relationship between primary tumor site and BCSM (P<0.0001; Table 2) was observed, whereas laterality had no effect on BCSM (P = 0.331; Table 2). Similar results could be obtained after stratification by the stage and tumor aggressiveness as potential confounders (Table S1 and S2). In addition, young age, early year of diagnosis, large tumor size, LN involvement, negative ER and PgR status, incomplete radiotherapy, and the inner and lower primary tumor sites were independently associated with increased BCSM (P<0.0001; Table 2).

**Table 2:** Multivariate Analysis of Breast Cancer-Specific Mortality (BCSM).

Variable	BCSM		Pairwise*	
	HR [95% CI]	P_1_ value	HR [95% CI]	P_2_ value
**Age of Diagnosis**				
**20–39**	1.000 [Reference]			
**40–59**	0.784 [0.752–0.818]	<.0001		
**60–79**	0.964 [0.924–1.007]	.098		
**Year of Diagnosis**				
**1990–1994**	1.000 [Reference]			
**1995–1999**	0.817 [0.788–0.847]	<.0001		
**2000–2004**	0.681 [0.657–0.705]	<.0001		
**2005–2009**	0.570 [0.545–0.597]	<.0001		
**Tumor Size**				
**0–2 cm**	1.000 [Reference]			
**2–5 cm**	2.286 [2.223–2.350]	<.0001		
**>5 cm**	3.714 [3.551–3.885]	<.0001		
**LN Status**				
**Negative**	1.000 [Reference]			
**Positive**	2,811 [2.736–2.888]	<.0001		
**ER Status**				
**Negative**	1.000 [Reference]			
**Positive**	0.623 [0.601–0.645]	<.0001		
**PR Status**				
**Negative**	1.000 [Reference]			
**Positive**	0.716 [0.691–0.741]	<.0001		
**Radiotherapy**				
**Without RT**	1.000 [Reference]			
**With RT**	0.892 [0.870–0.915]	<.0001		
**Laterality**				
**Left-sided**	1.000 [Reference]			
**Right-sided**	0.988 [0.964–1.013]	.331		
**Primary Site**		<.0001		
**UO**	1.000 [Reference]		0.920 [0.880–0.961	<.0001
**UI**	1.242 [1.199–1.287]	<.0001	1.142 [1.085–1.203]	<.0001
**LI**	1.329 [1.270–1.391]	<.0001	1.222 [1.152–1.296]	<.0001
**LO**	1.137 [1.091–1.185]	<.0001	1.046 [0.989–1.105]	.117
**CEN**	1.087 [1.041–1.136]	<.0001	1.000 [Reference]	

Abbreviations: HR =  hazard ratio; CI =  confidence interval; AJCC =  American Joint Committee on Cancer; LN =  lymph node; ER =  estrogen receptor; PR =  progesterone receptor; RT =  radiotherapy; UO =  upper outer quadrant of breast; UI =  upper inner quadrant of breast; LI =  lower inner quadrant of breast; LO =  lower outer quadrant of breast; CEN =  central portion quadrant of breast.

*Pairwise comparisons were conducted to clarify the difference on HR at different primary site. P_1_ value, using UO as the reference, indicated best prognosis for patients with tumors at UO. From another point of view, P_2_ value, using CEN as the reference, indicated that patients with tumors at CEN and LO had worse prognosis than those at UO, but better prognosis than those at UI and LI.

### The Effect of Primary Tumor Site on BCSM after Stratification by Laterality

**Figure 3:**
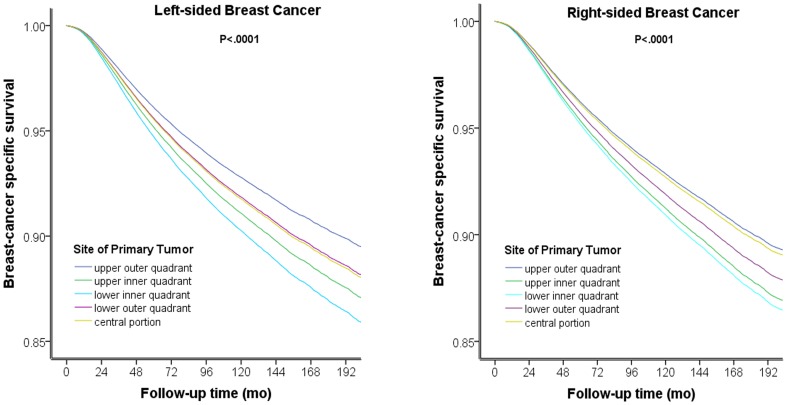
Survival Curves Stratified According to the Primary Tumor Site for Breast Cancer. The survival curves stratified according to primary tumor site for both sides indicate that primary tumors on the left and right side display different effects BCSM. For left-side breast cancer, patients with central portion tumors had a similar prognosis as patients with lower-outer quadrant tumors (A). For right-sided breast cancer, patients with central portion tumors displayed survival similar to patients with upper-outer quadrant tumors (B).

We stratified patients according to laterality and then compared the contribution of primary tumor site to BCSM for each side. Using the upper-outer quadrant as the reference, the HR was increased in the central portion as well as the lower-outer, upper-inner and lower-inner quadrants (HRs from 1.135–1.369, P<0.0001, Table S3) for patients with left-sided breast cancer; patients with tumors in the central portion had the same prognosis as patients with tumors in the lower-outer quadrant (P = 0.764; Table S3). However, right-sided breast cancer presented with a different HR rank compared with left-sided breast cancer (using the upper-outer quadrant as the reference, central portion: P = 0.471; lower-outer quadrant: HR = 1.141, 95% CI = 1.074–1.211, P<0.0001; upper-inner quadrant: HR = 1.238, 95% CI = 1.176–1.303, P<0.0001; lower-inner quadrant: HR = 1.284, 95% CI = 1.200–1.374, P<0.0001; Table S4). Patients with tumors in the central portion experienced similar survival compared with patients with tumors in the upper-outer quadrant (P = 0.471; Table S4). The survival curves stratified according to primary tumor site on both sides are presented in Figure 3, indicating that left and right primary tumor sites have different effects on BCSM.

### The Combined Effect of Laterality and Primary Tumor Site on BCSM

In addition, we combined laterality with primary tumor site to specify the contribution of both factors to BCSM. Tumors on the right upper-outer quadrant (P = 0.755; Table 3) and right central portion (P = 0.329; Table 3) exhibited no difference compared with tumors at the left upper-outer quadrant. Tumors at the left central portion and the left lower-outer and right lower-outer quadrants displayed slightly increased HRs compared with the left upper-outer quadrant (HRs from 1.136 to 1.145, P<0.0001; Table 3), whereas tumors located in the inner quadrants displayed increased HRs (HRs from 1.242 to 1.372, P<0.0001; Table 3).

**Table 3:** Comparison of BCSM Using Combination of Laterality with Primary Tumor Site.

Specific Location	BCSM		Pairwise*	
	HR [95% CI]	P_1_ value	HR [95% CI]	P_2_ value
**LUO**	1.000 [Reference]		0.968 [0.908–1.033]	.329
**LUI**	1.250 [1.189–1.313]	<.0001	1.210 [1.124–1.303]	<.0001
**LLI**	1.372 [1.290–1.459]	<.0001	1.329 [1.233–1.443]	<.0001
**LLO**	1.136 [1.072–1.203]	<.0001	1.100 [1.016–1.191]	.019
**LCEN**	1.144 [1.078–1.215]	<.0001	1.108 [1.022–1.202]	.013
**RUO**	1.005 [0.972–1.039]	.755	0.974 [0.913–1.038]	.415
**RUI**	1.242 [1.180–1.307]	<.0001	1.202 [1.115–1.297]	<.0001
**RLI**	1.288 [1.204–1.377]	<.0001	1.247 [1.143–1.360]	<.0001
**RLO**	1.145 [1.079–1.216]	<.0001	1.109 [1.022–1.203]	.013
**RCEN**	1.033 [0.968–1.101]	.329	1.000 [Reference]	

Abbreviations: BCSM =  breast cancer-specific mortality; HR =  hazard ratio; CI =  confidence interval; LUO =  left upper outer quadrant of breast; LUI =  left upper inner quadrant of breast; LLI =  left lower inner quadrant of breast; LLO =  left lower outer quadrant of breast; LCEN =  left central portion quadrant of breast; RUO =  right upper outer quadrant of breast; RUI =  right upper inner quadrant of breast; RLI =  right lower inner quadrant of breast; RLO =  right lower outer quadrant of breast; RCEN =  right central portion quadrant of breast.

*Pairwise comparisons were conducted to clarify the difference on HR at different specific location. P_1_ value, using LUO as the reference, indicated same prognosis for patients with tumors at LUO, RUO and RCEN which were better than any other location. From another point of view, P_2_ value, using RCEN as the reference, indicated a little bit worse prognosis for patients with tumors at LCEN, LLO, RLO and the poorest prognosis for patients with tumors at LUI, LLI, RUI, RLI.

Moreover, we evaluated whether the primary tumor site and laterality interacted to contribute to BCSM. With respect to overall patients, no significant interaction between laterality and primary tumor site was observed (P = 0.109; Table S5), with the exception of tumors located in the central portion (HR = 0.898, 95% CI = 0.822–0.980, P = 0.016; Table S5), which was predicted by the Cox regression model.

## Discussion

Our study suggests that BCSM is influenced by primary tumor site but not by laterality respectively. However, if the combined effect of laterality and primary tumor site on BCSM is taken into consideration, tumors located in the upper-outer quadrant on either side or the right central portion have the best prognosis, whereas tumors in the lower-outer quadrant on either side or the left central portion are at increased risk. Tumors in the inner region on both sides display the poorest results. Besides, subgroup analysis indicates that laterality contributes to BCSM only for central portion tumors.

In our study, breast tumors in the lower and inner quadrants tend to display poorer prognoses distinctly on either side in accordance with previous studies on the overall effect [4]–[6]. We hypothesize its relation to the drainage of internal and inferior mammary lymph nodes. Increased IMC drainage was observed in breast tumors in the lower-inner quadrant compared with tumors at other sites [11]. IMC drainage on preoperative lymphoscintigraphy was significantly associated with poorer distant disease-free survival (DDFS) but not overall survival or local-regional recurrence [12]. Based on these previous studies, we can explain why patients with tumors in the lower-inner quadrant had elevated BCSM with regard to the following three factors: clinical examination, imaging detection, and anatomic characteristics. The current clinical breast examination covers the entire breast as well as axillary and supraclavicular lymph nodes; thus, medial and inferior lymphatic groups are not included. Similar problems are observed in imaging detection because it's relatively difficult to discover abnormalities in the internal mammary region via technical manipulation. In addition, internal mammary lymph node drainage can be involved with contralateral breast cancer; hepatic metastasis can occur through inferior mammary lymph node drainage, which underscores the tendency of lower-inner quadrant tumors to display distant metastasis. Thus, compared with axillary lymph nodes, internal and inferior mammary lymph nodes could not be easily detected and more associated with severe cases, which is in accordance with the result that patients with tumors in lower and inner quadrant have a poorer prognosis than those with tumors in upper and outer quadrant.

With regard to the differing effect of laterality on BCSM in central portion tumors, we hypothesize that different lymph drainage patterns may exist for central portion breast tumors on different sides. Lymphoscintigraphy was once used to detect the lymph drainage patterns from different breast quadrants, but laterality was not included as a category standard [11]–[13]. Further laterality studies can be performed to determine whether breast tumors located in the central portion on the left side display increased lymph drainage to IMC compared with tumors on the right side. However, left-right asymmetry in embryonic development cannot be neglected. A possible correlation was observed between the molecular control of laterality and cancer predisposition [14]; however, the study must be confirmed. We hope that specific molecules will be used to research the relationship between left-right asymmetry and breast cancer.

Our study has several limitations which are mainly focused on the section that laterality contributes to BCSM only for central portion tumors. First, the sample size in the central group is relatively insufficient compared with the entire sample size, which may lead to the false positive significance in the effect of laterality on BCSM for central portion tumors, because on the level of entire sample size, laterality is not an overall indicator of breast cancer-specific survival in our study in accordance with previous studies [15]–[17]. Second, the suppositions of different lymph drainage patterns and left-right asymmetry in embryonic development are not so persuasive to explain the difference of BCSM for central portion tumors on different sides. Further studies are needed to verify the hypotheses. Third, we have once thought the possibilities of association between handedness and laterality of breast cancer. But it cannot be judged due to the lack of specific information about handedness could be got in SEER Database.

Our study demonstrates that the primary tumor site should serve as a prognostic factor in breast cancer. More attention should be given to tumors located at the inner region on both sides. Moreover, we hope further studies will determine the mechanism explaining the differing effects of tumor location on BCSM.

## Supporting Information

Table S1 **Multivariate Analysis of BCSM Stratified by the Stage of Breast Cancer.**
(DOCX)

Table S2 **Multivariate Analysis of BCSM Stratified by the Grade of Breast Cancer.**
(DOCX)

Table S3 **Multivariate Analysis of BCSM for Left-sided Breast Cancer.**
(DOCX)

Table S4 **Multivariate Analysis of BCSM for Right-sided Breast Cancer.**
(DOCX)

Table S5 **Interaction between Laterality and Primary Tumor Site for the Contribution to BCSM.**
(DOCX)
