# Supplementary material for: The Effect of Laterality and Primary Tumor Site on Cancer-Specific Mortality in Breast Cancer: A SEER Population-Based Study
Source: PLoS One. 2014 Apr 16;9(4):e94815. doi: 10.1371/journal.pone.0094815 (PMC3989248; doi:10.1371/journal.pone.0094815)
Supplement: Table S1 — Multivariate Analysis of BCSM Stratified by the Stage of Breast Cancer. (DOCX) [file pone.0094815.s001.docx]

**Table S1.** Multivariate Analysis of BCSM Stratified by the Stage of Breast Cancer

| Variable | BCSM | |
| --- | --- | --- |
|  | HR [95% CI] | P_1_ value |
| Laterality (Stage I) |  |  |
| Left-sided | 1.000 [Reference] |  |
| Right-sided | 0.994 [0.943-1.048] | .824 |
| Primary Site (Stage I) |  | <.0001 |
| UO | 1.000 [Reference] |  |
| UI | 1.254 [1.170-1.343] | <.0001 |
| LI | 1.263 [1.158-1.378] | <.0001 |
| LO | 1.192 [1.091-1.302] | <.0001 |
| CEN | 1.099 [0.991-1.219] | .073 |
| Laterality (Stage II) |  |  |
| Left-sided | 1.000 [Reference] |  |
| Right-sided | 0.980 [0.944-1.016] | .274 |
| Primary Site (Stage II) |  | <.0001 |
| UO | 1.000 [Reference] |  |
| UI | 1.263 [1.200-1.329] | <.0001 |
| LI | 1.374 [1.287-1.467] | <.0001 |
| LO | 1.100 [1.034-1.170] | .002 |
| CEN | 1.109 [1.037-1.185] | .002 |
| Laterality (Stage III) |  |  |
| Left-sided | 1.000 [Reference] |  |
| Right-sided | 0.993 [0.950-1.037] | .740 |
| Primary Site (Stage III) |  | <.0001 |
| UO | 1.000 [Reference] |  |
| UI | 1.124 [1.045-1.208] | .002 |
| LI | 1.162 [1.060-1.275] | .001 |
| LO | 1.026 [0.954-1.103] | .486 |
| CEN | 1.125 [1.048-1.208] | .001 |

Abbreviations: HR = hazard ratio; CI = confidence interval; UO = upper outer quadrant of breast; UI = upper inner quadrant of breast; LI = lower inner quadrant of breast; LO = lower outer quadrant of breast; CEN = central portion quadrant of breast.
