# Supplementary material for: The Effect of Laterality and Primary Tumor Site on Cancer-Specific Mortality in Breast Cancer: A SEER Population-Based Study
Source: PLoS One. 2014 Apr 16;9(4):e94815. doi: 10.1371/journal.pone.0094815 (PMC3989248; doi:10.1371/journal.pone.0094815)
Supplement: Table S2 — Multivariate Analysis of BCSM Stratified by the Grade of Breast Cancer. (DOCX) [file pone.0094815.s002.docx]

**Table S2.** Multivariate Analysis of BCSM Stratified by the Grade of Breast Cancer

| Variable | BCSM | |
| --- | --- | --- |
|  | HR [95% CI] | P_1_ value |
| Laterality (Grade I) |  |  |
| Left-sided | 1.000 [Reference] |  |
| Right-sided | 1.074 [0.958-1.203] | .220 |
| Primary Site (Grade I) |  | .012 |
| UO | 1.000 [Reference] |  |
| UI | 1.265 [1.130-1.393] | .001 |
| LI | 1.387 [1.145-1.679] | .001 |
| LO | 1.101 [0.908-1.334] | .329 |
| CEN | 1.010 [0.826-1.235] | .923 |
| Laterality (Grade II) |  |  |
| Left-sided | 1.000 [Reference] |  |
| Right-sided | 1.008 [0.963-1.055] | .730 |
| Primary Site (Grade II) |  | <.0001 |
| UO | 1.000 [Reference] |  |
| UI | 1.250 [1.170-1.335] | <.0001 |
| LI | 1.315 [1.211-1.428] | <.0001 |
| LO | 1.129 [1.046-1.218] | .002 |
| CEN | 1.145 [1.060-1.236] | .001 |
| Laterality (Grade III) |  |  |
| Left-sided | 1.000 [Reference] |  |
| Right-sided | 0.958 [0.926-1.002] | .050 |
| Primary Site (Grade III) |  | <.0001 |
| UO | 1.000 [Reference] |  |
| UI | 1.220 [1.162-1.281] | <.0001 |
| LI | 1.289 [1.211-1.373] | <.0001 |
| LO | 1.081 [1.021-1.145] | .007 |
| CEN | 1.106 [1.037-1.178] | .002 |

Abbreviations: HR = hazard ratio; CI = confidence interval; UO = upper outer quadrant of breast; UI = upper inner quadrant of breast; LI = lower inner quadrant of breast; LO = lower outer quadrant of breast; CEN = central portion quadrant of breast.
