# Supplementary material for: The Effect of Laterality and Primary Tumor Site on Cancer-Specific Mortality in Breast Cancer: A SEER Population-Based Study
Source: PLoS One. 2014 Apr 16;9(4):e94815. doi: 10.1371/journal.pone.0094815 (PMC3989248; doi:10.1371/journal.pone.0094815)
Supplement: Table S3 — Multivariate Analysis of BCSM for Left-sided Breast Cancer. (DOCX) [file pone.0094815.s003.docx]

**Table S3.** Multivariate Analysis of BCSM for Left-sided Breast Cancer

| Variable | BCSM | | Pairwise | | |
| --- | --- | --- | --- | --- | --- |
|  | HR [95% CI] | P value | HR [95% CI] | P value |  |
| Age of Diagnosis |  |  |  |  |  |
| 20-39 | 1.000 [Reference] |  |  |  |  |
| 40-59 | 0.803 [0.757-0.851] | <.0001 |  |  |  |
| 60-79 | 0.988 [0.930-1.049] | .693 |  |  |  |
| Year of Diagnosis |  | <.0001 |  |  |  |
| 1990-1994 | 1.000 [Reference] |  |  |  |  |
| 1995-1999 | 0.804 [0.765-0.846] |  |  |  |  |
| 2000-2004 | 0.681 [0.648-0.714] |  |  |  |  |
| 2005-2009 | 0.574 [0.539-0.611] |  |  |  |  |
| Tumor Size |  | <.0001 |  |  |  |
| 0-2cm | 1.000 [Reference] |  |  |  |  |
| 2-5cm | 2.301 [2.213-2.392] |  |  |  |  |
| >5cm | 3.630 [3.408-3.867] |  |  |  |  |
| LN Status |  | <.0001 |  |  |  |
| Negative | 1.000 [Reference] |  |  |  |  |
| Positive | 2.800 [2.697-2.908] |  |  |  |  |
| ER Status |  | <.0001 |  |  |  |
| Negative | 1.000 [Reference] |  |  |  |  |
| Positive | 0.613 [0.584-0.644] |  |  |  |  |
| PR Status |  | <.0001 |  |  |  |
| Negative | 1.000 [Reference] |  |  |  |  |
| Positive | 0.711 [0.678-0.746] |  |  |  |  |
| Radiotherapy |  | <.0001 |  |  |  |
| Without RT | 1.000 [Reference] |  |  |  |  |
| With RT | 0.903 [0.872-0.936] |  |  |  |  |
| Primary site |  | <.0001 |  |  |  |
| UO | 1.000 [Reference] |  | 0.871 [0.820-0.925] | <.0001 |  |
| UI | 1.247 [1.187-1.311] |  | 1.086 [1.012-1.166] | .021 |  |
| LI | 1.369 [1.287-1.456] |  | 1.192 [1.101-1.291] | <.0001 |  |
| LO | 1.135 [1.072-1.202] |  | 0.988 [0.916-1.067] | .764 |  |
| CEN | 1.148 [1.081-1.219] |  | 1.000 [Reference] |  |  |

Abbreviations: HR = hazard ratio; CI = confidence interval; LN = lymph node; ER = estrogen receptor; PR = progesterone receptor; RT= radiotherapy; UO = upper outer quadrant of breast; UI = upper inner quadrant of breast; LI = lower inner quadrant of breast; LO = lower outer quadrant of breast; CEN = central portion quadrant of breast.
