# Supplementary material for: The Effect of Laterality and Primary Tumor Site on Cancer-Specific Mortality in Breast Cancer: A SEER Population-Based Study
Source: PLoS One. 2014 Apr 16;9(4):e94815. doi: 10.1371/journal.pone.0094815 (PMC3989248; doi:10.1371/journal.pone.0094815)
Supplement: Table S4 — Multivariate Analysis of BCSM for Right-sided Breast Cancer. (DOCX) [file pone.0094815.s004.docx]

**Table S4.** Multivariate Analysis of BCSM for Right-sided Breast Cancer

| Variable | BCSM | | Pairwise | | |
| --- | --- | --- | --- | --- | --- |
|  | HR [95% CI] | P value | HR [95% CI] | P value |  |
| Age of Diagnosis |  |  |  |  |  |
| 20-39 | 1.000 [Reference] |  |  |  |  |
| 40-59 | 0.765 [0.721-0.813] | <.0001 |  |  |  |
| 60-79 | 0.940 [0.883-0.999] | .048 |  |  |  |
| Year of Diagnosis |  | <.0001 |  |  |  |
| 1990-1994 | 1.000 [Reference] |  |  |  |  |
| 1995-1999 | 0.831 [0.789-0.875] |  |  |  |  |
| 2000-2004 | 0.681 [0.647-0.716] |  |  |  |  |
| 2005-2009 | 0.567 [0.531-0.605] |  |  |  |  |
| Tumor Size |  | <.0001 |  |  |  |
| 0-2cm | 1.000 [Reference] |  |  |  |  |
| 2-5cm | 2.271 [2.182-2.364] |  |  |  |  |
| >5cm | 3.802 [3.565-4.054] |  |  |  |  |
| LN Status |  | <.0001 |  |  |  |
| Negative | 1.000 [Reference] |  |  |  |  |
| Positive | 2.821 [2.714-2.933] |  |  |  |  |
| ER Status |  | <.0001 |  |  |  |
| Negative | 1.000 [Reference] |  |  |  |  |
| Positive | 0.634 [0.602-0.667] |  |  |  |  |
| PR Status |  | <.0001 |  |  |  |
| Negative | 1.000 [Reference] |  |  |  |  |
| Positive | 0.721 [0.686-0.757] |  |  |  |  |
| Radiotherapy |  | <.0001 |  |  |  |
| Without RT | 1.000 [Reference] |  |  |  |  |
| With RT | 0.881 [0.850-0.914] |  |  |  |  |
| Primary site |  | <.0001 |  |  |  |
| UO | 1.000 [Reference] |  | 0.977 [0.915-1.042] | .471 |  |
| UI | 1.238 [1.176-1.303] | <.0001 | 1.208 [1.120-1.303] | <.0001 |  |
| LI | 1.284 [1.200-1.374] | <.0001 | 1.254 [1.149-1.368] | <.0001 |  |
| LO | 1.141 [1.074-1.211] | <.0001 | 1.114 [1.027-1.209] | <.0001 |  |
| CEN | 1.024 [0.960-1.092] | .471 | 1.000 [Reference] |  |  |

Abbreviations: HR = hazard ratio; CI = confidence interval; LN = lymph node; ER = estrogen receptor; PR = progesterone receptor; RT= radiotherapy; UO = upper outer quadrant of breast; UI = upper inner quadrant of breast; LI = lower inner quadrant of breast; LO = lower outer quadrant of breast; CEN = central portion quadrant of breast.
