# Supplementary material for: The Effect of Laterality and Primary Tumor Site on Cancer-Specific Mortality in Breast Cancer: A SEER Population-Based Study
Source: PLoS One. 2014 Apr 16;9(4):e94815. doi: 10.1371/journal.pone.0094815 (PMC3989248; doi:10.1371/journal.pone.0094815)
Supplement: Table S5 — Interaction between Laterality and Primary Tumor Site for the Contribution to BCSM. (DOCX) [file pone.0094815.s005.docx]

**Table S5.** Interaction between Laterality and Primary Tumor Site for the Contribution to BCSM

| Variable | BCSM | |
| --- | --- | --- |
|  | HR [95% CI] | P value |
| Laterality*Site of primary tumor |  | .109 |
| LAT*UO | 1.000 [Reference] |  |
| LAT*UI | 0.988 [0.921-1.061] | .745 |
| LAT*LI | 0.934 [0.852-1.022] | .138 |
| LAT*LO | 1.003 [0.923-1.090] | .937 |
| LAT*CEN | 0.898 [0.822-0.980] | .016 |

Abbreviations: HR = hazard ratio; CI = confidence interval; LN = lymph node; ER = estrogen receptor; PR = progesterone receptor; RT= radiotherapy; UO = upper outer quadrant of breast; UI = upper inner quadrant of breast; LI = lower inner quadrant of breast; LO = lower outer quadrant of breast; CEN = central portion quadrant of breast, LAT = laterality.
